# Polystyrene microplastics impair the function of human retinal microvascular endothelial cells and pericytes and increase vascular permeability *in vitro*

**DOI:** 10.3389/fmed.2025.1587759

**Published:** 2025-06-24

**Authors:** Jang-Hyuk Yun

**Affiliations:** College of Veterinary Medicine and Institute of Veterinary Science, Kangwon National University, Chuncheon, Republic of Korea

**Keywords:** polystyrene, pericyte, endothelial cell, endothelial permeability, retinopathy

## Abstract

**Introduction:**

Polystyrene (PS) microplastics are among the most prevalent types of microplastics responsible for global pollution. Although numerous studies have investigated the effects of PS on various organs, such as the heart, lungs, liver, kidneys, nervous system, and intestines, its impact on the eyes, particularly the retina, remains largely unexplored.

**Methods:**

To assess the effects of PS on retinal pathology, cultured retinal microvascular endothelial cells, pericytes, astrocytes, and microglial cells were exposed to 2 μm PS particles. Cell viability (MTT assay), apoptosis (Annexin V/PI flow cytometry), protein expression (Western blotting), and angiogenesis-related behaviors (tube formation, migration, and permeability assays) were evaluated.

**Results:**

PS induced endothelial cell apoptosis by reducing the activity of AKT and ERK1/2, and induced pericyte apoptosis by reducing the activity of AKT. PS also impaired tube formation, migration, and proliferation by reducing AKT and ERK1/2 activity in retinal endothelial cells. In addition, PS induced pericyte apoptosis and increased endothelial permeability.

**Conclusion:**

PS may worsen retinopathy by inducing endothelial cell and pericyte apoptosis and by increasing vascular leakage, although it does not promote angiogenesis.

## 1 Introduction

Microplastics are emerging contaminants found extensively in oceans, freshwater, soil, and air and have significant impacts on human life (1). When ingested by marine and terrestrial organisms, microplastics accumulate in tissues and organs, causing high levels of damage as they move through food chains. Recently, microplastics have been detected in various foods, including seafood, sea salt, and drinking water (2–6), as well as in the human placenta, feces, sputum, and blood (7–10). These findings suggest that microplastics can infiltrate and accumulate in the human body, potentially posing risks to multiple organ systems.

Polystyrene (PS) microplastics, frequently used in food packaging and disposable takeout containers, are among the most prevalent microplastic components (11, 12). PS has been extensively studied due to its deleterious effects. Numerous studies demonstrated that PS can cause toxicity in various organs, including the heart, lungs, liver, kidneys, digestive tract, and nervous system of experimental animals, through different exposure routes (13–15). Interestingly, when PS is orally administered to mice, it increases the permeability of the blood–brain barrier and accumulates in the mouse brain (16). Similar to the blood–brain barrier, the retina is protected by the blood–retinal barrier (BRB), which functions as a neurovascular unit composed of endothelial cells with tight junctions, a dense pericyte layer, and astrocyte end-feet (17, 18). Many substances that pass through the blood–brain barrier also pass through the BRB (19). In addition, orally administered PS can reach the retina in mice (20). However, little is known about the effects of PS on the retina.

Retinopathy is caused by various factors, notably diabetic retinopathy, which results from high blood sugar levels (21), and retinopathy of prematurity, which is associated with premature births (22). Diabetic retinopathy is characterized by damage to the capillaries, abnormal angiogenesis, and vascular leakage in the retina, leading to visual impairment and blindness in working-age individuals (23). In contrast, retinopathy of prematurity is caused by abnormal angiogenesis in the retina and can result in blindness in children (22). In addition, administration of oxygen to treat hypoxia in premature infants can damage the retinal microvasculature, potentially worsening retinopathy of prematurity (22). Damage to retinal blood vessels, abnormal angiogenesis, or vascular leakage can induce or exacerbate retinopathy. However, the effects of PS on abnormal angiogenesis and vascular leakage in the retina are not well understood.

Endothelial cells and pericytes play key roles in abnormal angiogenesis and vascular leakage. The proliferation, migration, and tube formation of endothelial cells represent the multistep process of angiogenesis (24, 25). An increase or decrease in the expression of tight junction proteins in endothelial cells indicates a decrease or increase in vascular leakage, respectively (26, 27). Pericytes can inhibit angiogenesis in proliferating endothelial cells, a process that tends to occur in regions of pericyte loss (28). In addition, pericytes reduce endothelial permeability by enhancing the expression of tight junction proteins, thereby preventing vascular leakage, which increases when pericyte apoptosis occurs (27). However, the direct effect of PS on endothelial cells and pericytes remains unclear. Therefore, we examined these related areas.

These findings revealed that PS was capable of inducing apoptosis in retinal endothelial cells and reducing their proliferation, migration, and tube formation by reducing AKT and ERK1/2 activity. Furthermore, PS triggers apoptosis in pericytes by inhibiting AKT activity, which in turn weakens the tight junctions of endothelial cells in the co-culture of pericytes and endothelial cells, thereby increasing endothelial permeability. These findings suggest that PS contributes to or exacerbates retinopathy symptoms associated with vascular leakage via pericyte apoptosis, although it does not aggravate angiogenesis.

## 2 Materials and methods

### 2.1 Reagents and antibodies

PS (spherical, 2 μm diameter, 10% solids suspension in sterile distilled water), 3-(4,5-dimethylthiazol-2-yl)-2,5-diphenyltetrazolium bromide (MTT), ceramide C6, Evans blue dye, and bovine serum albumin were purchased from Millipore Sigma (St. Louis, MO, USA). PS particles were suspended in sterile distilled water and sonicated for 30 min to minimize aggregation prior to use. SC79 was obtained from Selleck Chemicals (Houston, TX, USA). Other reagents and antibodies used were as follows: anti-cleaved caspase-3, anti-Bax, anti-Bcl-2, anti-Bcl-xL, anti-phospho-AKT, anti-AKT, anti-phospho-ERK1/2, and anti-ERK1/2 (Cell signaling Technology, Danvers, MA, USA); anti-β-tubulin and peroxidase-conjugated secondary antibodies (Santa Cruz Biotechnology, Dallas, TX, USA); anti-ZO-1 and anti-occludin (Thermo Fisher Scientific, Waltham, MA, USA); FITC-conjugated Annexin-V/PI assay kit (BD Biosciences, Franklin Lakes, NJ, USA); and 5′-bromodeoxy-2′-uridine (BrdU) cell proliferation enzyme-linked immunosorbent assays (Roche, Indianapolis, IN, USA).

### 2.2 Cell cultures

Human primary retinal microvascular endothelial cells (HRMECs; ACBRI, Kirkland, WA, USA) and human pericytes from the placenta (PromoCell, Heidelberg, Germany) were cultured in M199 medium (HyClone, Logan, UT, USA) containing 20% fetal bovine serum (FBS) and pericyte medium containing growth factors (PromoCell). The immortalized human microglial cell line HMO6, which has been characterized previously (27), was cultured in Dulbecco’s Modified Eagle Medium (Thermo Fisher Scientific) supplemented with 10% FBS. Human astrocytes (ACBRI) were grown in Dulbecco’s Modified Eagle Medium supplemented with 10% FBS. Cells were incubated at 37°C in a 5% CO_2_ atmosphere. Upon thawing, all cell lines were gradually adapted to the culture conditions and passaged at least twice before experimental use. Only cells between passages 3 and 8 were used to ensure consistency and cellular stability.

### 2.3 Cell viability assay

Cell viability was assessed using the MTT assay (Millipore Sigma, Cat. #M5655) as previously described (29). In brief, approximately 5 × 10^3^ cells per well were seeded into 96-well flat-bottom plates (Corning, Inc., Corning, NY, USA) and allowed to attach for 24 h in a humidified incubator at 37°C with 5% CO₂. The cells were then treated with the indicated concentrations of PS particles or control reagents for 48 h. After treatment, 10 μL of MTT solution (5 mg/mL in PBS) was added to each well, and the plates were incubated for an additional 3 h at 37°C. The resulting formazan crystals were dissolved by adding 100 μL of dimethyl sulfoxide (Millipore Sigma) per well, and absorbance was measured at 570 nm using a microplate reader.

### 2.4 Fluorescence-activated cell sorting analysis

To assess apoptosis, 5 × 10^5^ cells were seeded into 6-well plates and treated with the indicated reagents at 37°C in a humidified incubator with 5% CO₂ for 48 h. After treatment, both adherent and floating cells were collected, washed twice with cold PBS, and resuspended in 100 μL of 1 × assay buffer. Apoptosis was analyzed using a Muse Annexin V & Dead Cell Kit (Millipore Sigma) according to the manufacturer’s protocol (30). Samples were incubated with staining solution for 20 min at room temperature in the dark and analyzed using a Muse Cell Analyzer (Millipore Sigma). Data were processed using Muse Analysis Software, and cells positive for Annexin-V only or Annexin-V/PI double staining were considered apoptotic.

### 2.5 Western blot analysis

Cells were harvested and lysed in radioimmunoprecipitation assay buffer (Thermo Fisher Scientific) supplemented with a protease and phosphatase inhibitor cocktail (Thermo Fisher Scientific). The protein concentration was determined using the bicinchoninic acid assay (Thermo Fisher Scientific), and equal amounts of protein (30 μg per lane) were resolved on 10–12% sodium dodecyl sufate–polyacrylamide gels. Following electrophoresis, proteins were transferred onto nitrocellulose membranes (0.45 μm pore size; GE Healthcare, Chicago, IL, USA) using a semi-dry blotting system (Bio-Rad, Hercules, CA, USA). Membranes were blocked in 5% non-fat dry milk diluted in TBST buffer (Tris-buffered saline containing 0.1% Tween-20) for 1 h at room temperature. Primary antibodies were diluted 1:1,000 in 5% bovine serum albumin in TBST and incubated overnight at 4°C. The following antibodies were used: anti-cleaved caspase-3 (Cat. #9661), anti-Bax (Cat. #2772), anti-Bcl-2 (Cat. #15071), anti-Bcl-xL (Cat. #2764), anti-phospho-AKT (Ser473; Cat. #4060), anti-AKT (Cat. #9272), anti-phospho-ERK1/2 (Thr202/Tyr204; Cat. #4370), and anti-ERK1/2 (Cat. #4695), all from Cell Signaling Technology; anti-ZO-1 (Cat. #61–7,300) and anti-occludin (Cat. #71–1,500) from Thermo Fisher Scientific; and anti-β-tubulin (Cat. #sc-5274) from Santa Cruz Biotechnology. After three washes with TBST (10 min each), the membranes were incubated with horseradish peroxidase-conjugated anti-rabbit or anti-mouse secondary antibodies (Santa Cruz Biotechnology, diluted 1:5,000 in 5% non-fat dry milk/TBST) for 1 h at room temperature. Bands were visualized using enhanced chemiluminescence detection reagent (Thermo Fisher Scientific) and imaged using the ImageQuant LAS 500 system (GE Healthcare) as previously described by Mahmood and Yang (31).

### 2.6 Tube formation

To evaluate tube formation, 200 μL of growth factor-reduced Matrigel (Corning, Inc.) was evenly dispensed into each well of a 24-well plate and incubated at 37°C for 30 min to allow gel polymerization. Cells (5 × 10^4^) were resuspended in 500 μL of endothelial growth medium containing the indicated reagents and seeded onto the solidified Matrigel. After incubation for 24 h at 37°C in a humidified incubator with 5% CO₂, tubular structures were imaged using an inverted phase-contrast microscope. The tube length and total tube area were quantified using ImageJ software (NIH, Bethesda, MD, USA), as described by Arnaoutova and Kleinman (32).

### 2.7 Transwell cell migration assay

Cells were plated on the upper chambers of Transwell inserts with 8 μm pores and then inserted into 24-well plates. Next, 6 × 10^3^ cells were seeded into the upper chambers (Corning, Inc.) for 6 h and starved in a medium containing 1% FBS for 12 h. The cells were treated with the indicated agents and allowed to migrate through the pores for another 24 h. After incubation, non-migrated cells on the upper surface of the membrane were gently removed with a cotton swab. The membrane was washed with PBS and stained using Diff-Quik solution (Sysmex, Kobe, Japan) according to the manufacturer’s protocol. For quantitative analysis, cells at four randomly selected sites on one membrane were counted, and the values from the four different membranes were quantified, as previously described by Justus et al. (33).

### 2.8 BrdU enzyme-linked immunosorbent proliferation assay

Cell proliferation was assessed using a Cell Proliferation BrdU ELISA Kit (Roche) according to the manufacturer’s instructions. Cells were treated with PS in the presence or absence of the AKT activator SC79 or ERK activator ceramide C6 for 24 h. Following treatment, the cells were incubated with 10 μM BrdU labeling solution for 1 h at 37°C to allow incorporation into newly synthesized DNA. After fixation and incubation with a peroxidase-conjugated anti-BrdU antibody for 90 min at room temperature, the cells were washed to remove unbound antibodies. A substrate solution was added, and the reaction product was quantified by measuring absorbance at 450 nm using a microplate reader, as described by Porstmann et al. (34).

### 2.9 2Permeability assay

To establish an *in vitro* BRB model, Transwell inserts with 0.4 μm pore polyester membranes (Corning, Inc.) were inverted, and pericytes were seeded onto the bottom surface of the inserts at a density of 5 × 10^4^ cells/cm^2^. After incubation for 2 h at 37°C to allow attachment, the inserts were returned to the upright position, and HRMECs were seeded onto the top surface at 1 × 10^5^ cells/cm^2^. The co-cultures were then incubated for an additional 24 h to allow for monolayer formation. After stabilization, the cells were treated for 48 h with or without 2 μm PS particles, the AKT activator SC79, or the ERK activator ceramide C6. To assess endothelial permeability, Evans blue dye (0.1%) was added to the top chamber. After incubation, the optical density of the medium in the bottom chamber was measured at 650 nm using a microplate reader (Tecan, Männedorf, Switzerland), as previously described for *in vitro* BRB models using Transwell-based systems (35).

### 2.10 Statistical analysis

Statistical analyses were performed using GraphPad Prism software (GraphPad. Inc., La Jolla, CA, USA). Depending on the experimental design, unpaired two-tailed Student’s *t*-test (assuming unequal variances), one-way analysis of variance, or two-way analysis of variance followed by Tukey’s *post-hoc* test was used. A *p*-value of less than 0.05 was considered statistically significant. All quantitative data are presented as the mean ± standard deviation.

## 3 Results

### 3.1 PS induces apoptosis of retinal endothelial cells and pericytes

**Figure 1:**
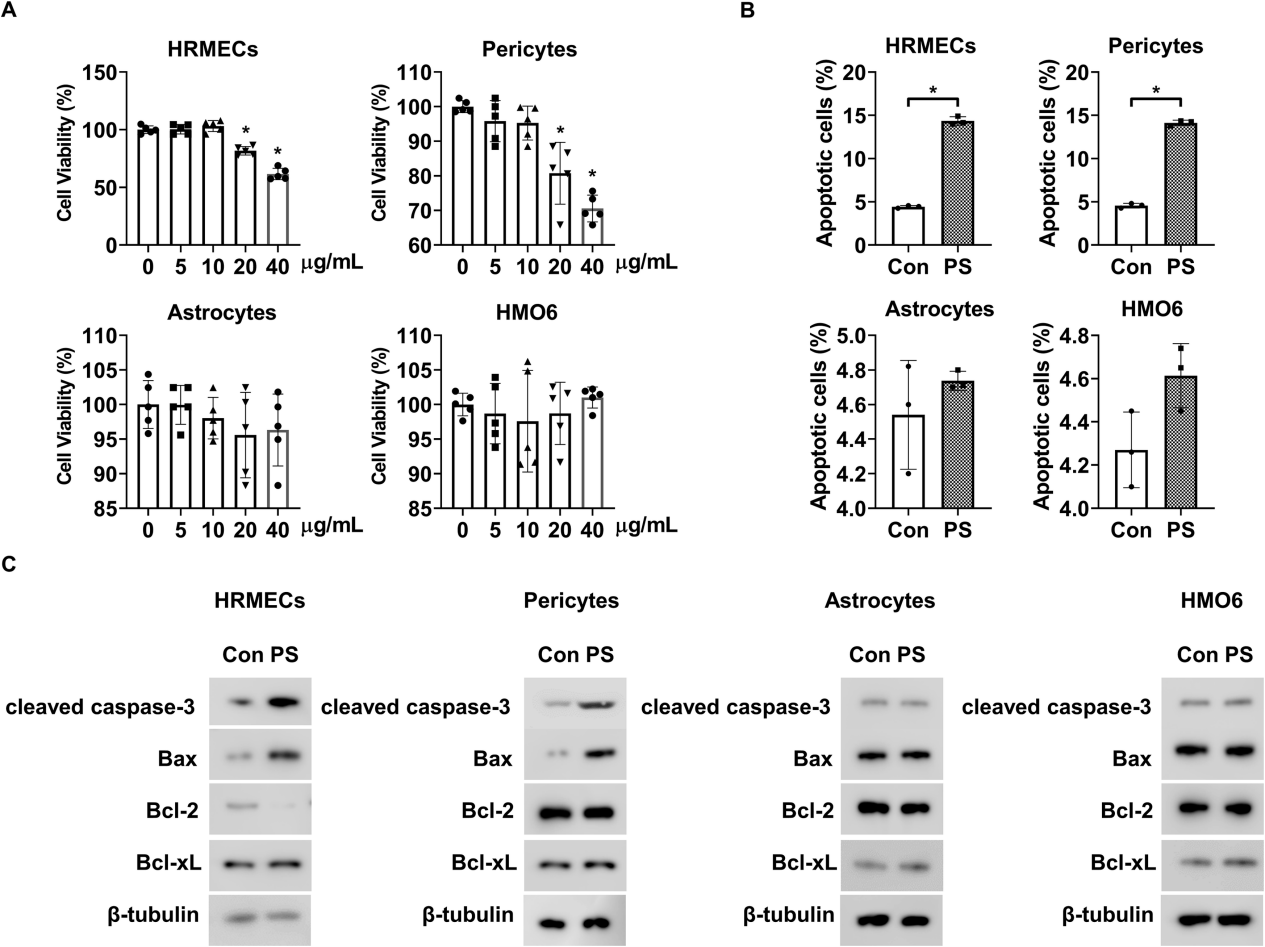
PS induces retinal apoptosis in retinal endothelial cells and pericytes. **(A)** HRMECs, pericytes, astrocytes, and HMO6 were treated with PS for 48 h at the indicated doses. Cell viability was analyzed via MTT assay. The bar graph shows the mean ± SD (*n* = 5). *p < 0.05, one-way ANOVA. **(B,C)** HRMECs, pericytes, astrocytes, and HMO6 were treated with PS (20 μg/mL) for 48 h, and **(B)** apoptosis was analyzed using Annexin-V/PI staining and flow cytometry. The bar graph shows the mean ± SD (*n* = 3). *p < 0.05, determined using Student’s *t*-test. **(C)** Western blot analysis was performed on lysates from HRMECs, pericytes, astrocytes, and HMO6 to detect cleaved caspase-3, Bax, Bcl-2, and Bcl-xL. *β*-tubulin was used as a loading control. ANOVA, analysis of variance; HMO6, human microglial cell line; HRMEC, human primary retinal microvascular endothelial cell; PS, polystyrene.

The MTT assay was used to determine the effect of PS on the viability of the four main cell types comprising BRBs: endothelial cells (HRMECs), pericytes, astrocytes, and microglia (HMO6). When the cells were exposed to PS for 48 h at the indicated doses, PS at doses higher than 10 ng/mL reduced the viability of HRMECs and pericytes but had no effect on the viability of astrocytes or HMO6 cells at all concentrations tested (Figure 1A).

Next, Annexin-V/PI flow cytometric and Western blot analyses were performed to confirm whether the PS-induced decrease in cell viability was related to apoptosis. PS induced apoptosis in HRMECs and pericytes but did not cause apoptosis in astrocytes or HMO6 cells (Figure 1B). PS increased cleaved caspase-3 and pro-apoptotic Bax levels and decreased anti-apoptotic Bcl-2 levels but did not alter pro-apoptotic Bcl-xL levels in HRMECs (Figure 1C; Supplementary Figure 1A). PS also increased cleaved caspase-3 and pro-apoptotic Bax levels in pericytes but did not change the levels of pro-apoptotic Bcl-2 and Bcl-xL (Figure 1C; Supplementary Figure 1B). Moreover, PS did not affect cleaved caspase-3, pro-apoptotic Bax, anti-apoptotic Bcl-2, or Bcl-xL levels in astrocytes or HMO6 cells (Figure 1C; Supplementary Figures 1C,D). These results indicate that PS induced apoptosis in HRMECs and pericytes.

### 3.2 PS triggers apoptosis in retinal endothelial cells by decreasing AKT and ERK1/2 activity and induces pericyte apoptosis by reducing AKT activity

**Figure 2:**
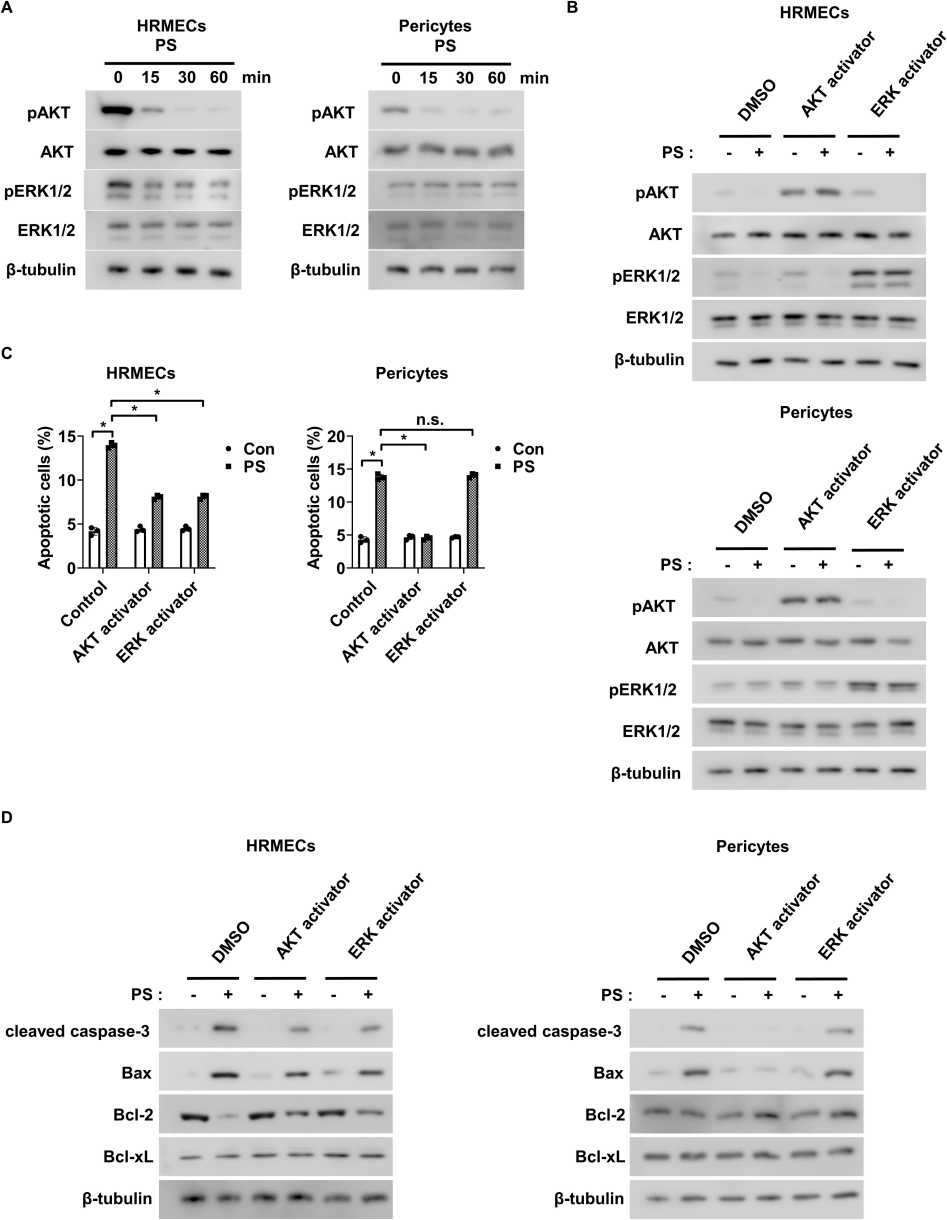
PS induces apoptosis by reducing AKT and ERK1/2 activity in retinal endothelial cells and decreasing AKT activity in pericytes. **(A)** Western blot analysis of pAKT, AKT, pERK1/2, and ERK1/2 was performed on lysates obtained from HRMECs or pericytes treated with PS (20 μg/mL) for the indicated times. β-tubulin was used as a loading control. **(B)** Western blot analysis of pAKT, AKT, pERK1/2, and ERK1/2 was performed on lysates obtained from HRMECs or pericytes preincubated with AKT activator SC79 (1 μg/mL) and ERK activator ceramide C6 (10 μM) for 1 h and then treated with PS (20 μg/mL) for 30 min. β-tubulin was used as a loading control. **(C,D)** HRMECs or pericytes were pretreated with AKT activator SC79 (1 μg/mL) and ERK activator ceramide C6 (10 μM) for 1 h and then treated with PS (20 μg/mL) for 48 h. **(C)** Cell apoptosis was analyzed via Annexin-V/PI staining and flow cytometry. The bar graph shows the mean ± SD (*n* = 3). n.s., not significant. *p < 0.05, two-way ANOVA. **(D)** Western blot analysis of cleaved caspase-3, Bax, Bcl-2, and Bcl-xL was performed on lysates obtained from HRMECs or pericytes. β-tubulin was used as a loading control. ANOVA, analysis of variance; HRMEC, human primary retinal microvascular endothelial cell; PS, polystyrene.

Next, the mechanism by which PS induces apoptosis in retinal endothelial cells and pericytes was investigated. Because AKT and ERK1/2 play roles in the survival of these cells (27, 36–38), we hypothesized that PS can trigger apoptosis by inhibiting their activity. PS reduced the phosphorylation of both AKT and ERK1/2 in a time-dependent manner in HRMECs, whereas it only reduced AKT phosphorylation in pericytes with no effect on ERK1/2 (Figure 2A; Supplementary Figure 2A). To explore the roles of AKT and ERK1/2 in PS-induced apoptosis, the AKT activator SC79 and ERK1/2 activator ceramide C6 were used. Both activators reversed the PS-induced decrease in AKT and ERK1/2 phosphorylation in HRMECs, and SC79 blocked the PS-induced decrease in AKT phosphorylation in pericytes (Figure 2B; Supplementary Figure 3A). Furthermore, SC79 and ceramide C6 both inhibited PS-induced apoptosis in HRMECs, whereas in pericytes, only SC79 was effective (Figure 2C). Both activators counteracted the PS-induced increase in cleaved caspase-3 and pro-apoptotic Bax levels, as well as the PS-induced decrease in anti-apoptotic Bcl-2 levels in HRMECs (Figure 2D; Supplementary Figure 4A). In pericytes, SC79 reduced the PS-induced increases in cleaved caspase-3 and Bax, whereas ceramide C6 did not (Figure 2D; Supplementary Figure 4B). These findings suggest that PS induces apoptosis by inhibiting both AKT and ERK1/2 activity in retinal endothelial cells and AKT activity in pericytes.

### 3.3 PS plays a role in suppressing angiogenesis

**Figure 3:**
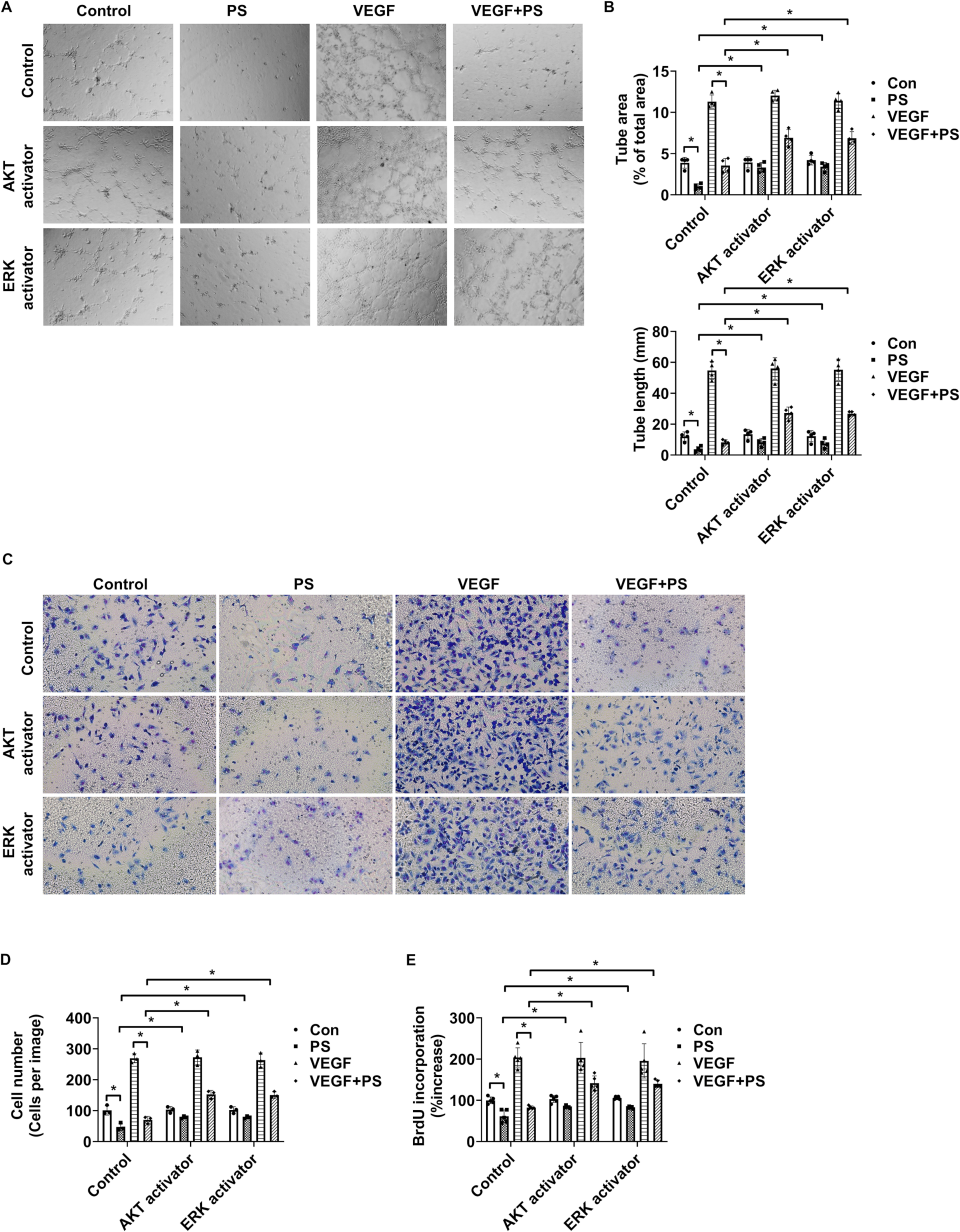
PS reduces retinal angiogenesis by decreasing AKT and ERK1/2 activity. **(A–E)** HRMECs were treated with VEGF (20 μg/mL), PS (20 μg/mL), AKT activator SC79 (1 μg/mL), and/or ERK activator ceramide C6 (10 μM) for 24 h. **(A)** Representative images of tube formation by HRMECs. Original magnification ×40. **(B)** Quantitative analysis of tube area (% of total area) and tube lengths (mm) in **(A)** was performed. The bar graph shows the mean ± SD (*n* = 4). *p < 0.05, two-way ANOVA with Tukey’s *post-hoc* test. **(C)** Representative images of cell migration of HRMECs. Migrated cells were stained with a Diff-Quik solution. Original magnification ×200. **(D)** Quantitative analysis of cell migration in **(C)** was performed. The bar graph represents the means ± SD (*n* = 3). *p < 0.05, two-way ANOVA with Tukey’s *post-hoc* test. **(E)** Cell proliferation was determined using the BrdU proliferation ELISA kit. The results are expressed as the percentage increase in BrdU incorporation versus control value. Mean ± SD (*n* = 5). *p < 0.05, two-way ANOVA with Tukey’s *post-hoc* test. ANOVA, analysis of variance; HRMEC, human primary retinal microvascular endothelial cell; PS, polystyrene.

Tube formation, cell migration, and cell proliferation assays were performed to investigate the effect of PS on angiogenesis. As AKT and ERK1/2 activation are closely related to angiogenesis induction in endothelial cells (39–41), the AKT activator SC79 and the ERK activator ceramide C6 were used to confirm the relationship between the decrease in AKT and ERK1/2 activation by PS and angiogenesis. First, the angiogenic effect of PS was analyzed by evaluating the tube-forming ability of retinal endothelial cells in Matrigel. PS alone reduced the area and the length of tube formation and significantly suppressed the tube formation area and the length of HRMECs increased by VEGF (Figures 3A,B). However, both SC79 and ceramide C6 significantly suppressed the PS-induced decrease in tube formation area and length (Figures 3A,B). To confirm the additional effect of PS on angiogenesis, the migration and proliferation of HRMECs were measured using Transwell and BrdU proliferation assays, respectively. PS reduced migration and proliferation even when used alone and significantly reduced the increase in migration and proliferation of HRMECs induced by VEGF (Figures 3C–E). However, both SC79 and ceramide C6 significantly inhibited the PS-induced decrease in cell migration and proliferation (Figures 3C–E). These results indicate that PS inhibits retinal angiogenesis by inhibiting the activity of AKT and ERK1/2.

### 3.4 PS increases endothelial permeability by inducing pericyte apoptosis

**Figure 4:**
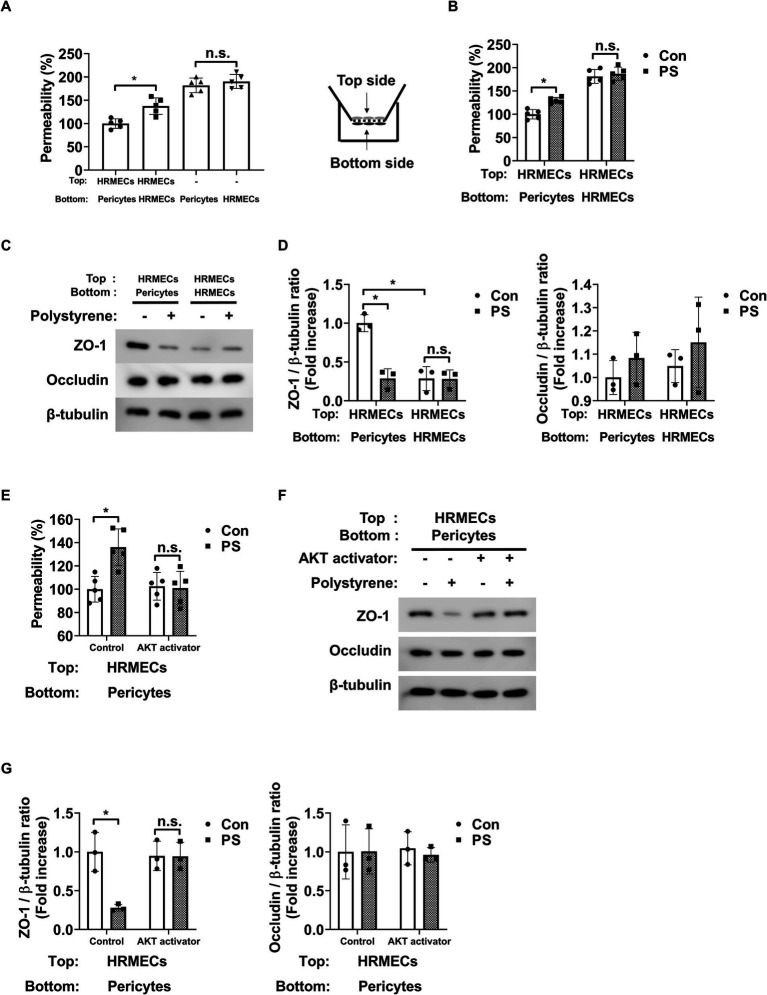
PS raises endothelial permeability by triggering apoptosis in pericytes. **(A)** Pericytes and HRMECs were co-cultured on the specified sides of the Transwell inserts as shown on the right. Permeability was assessed using Evans blue dye (*n* = 5). n.s., not significant. *p < 0.05, one-way ANOVA. **(B)** PS (20 μg/mL) was administered for 48 h under the indicated culture conditions, and permeability was subsequently measured (*n* = 5). n.s., not significant. *p < 0.05, two-way ANOVA. **(C)** Tight junction protein expression of ZO-1 and occludin was measured from the cell lysates of HRMECs on the top side of the Transwell obtained from (B). **(D)** Quantitative densitometric analysis in **(C)** to calculate the ratio of each protein to β-tubulin (*n* = 3). n.s., not significant. *p < 0.05, two-way ANOVA. **(E)** Permeability was measured after treatment with PS with or without AKT activator SC79 (1 μg/mL) in the co-cultured state of pericytes (Bottom) and HRMECs (Top) (*n* = 5). n.s., not significant. *p < 0.05, two-way ANOVA. **(F)** Tight junction protein expression of ZO-1 and occludin was measured from the cell lysates of HRMECs on the top side of the Transwell obtained from **(E)**. **(G)** Quantitative densitometric analysis in **(F)** to calculate the ratio of each protein to β-tubulin (*n* = 3). n.s., not significant. *p < 0.05, two-way ANOVA. ANOVA, analysis of variance; HRMEC, human primary retinal microvascular endothelial cell; PS, polystyrene.

To study the impact of PS on endothelial permeability, changes in the permeability of endothelial cells co-cultured with HRMECs or pericytes were assessed. When HRMECs were co-cultured with pericytes on both sides of the Transwell insert, their permeability decreased compared to cultures with only HRMECs (Figure 4A). This suggests that pericytes play a key role in reducing endothelial cell permeability. In addition, PS increased endothelial permeability when both pericytes and HRMECs were co-cultured on opposite sides of the Transwell membrane; however, this effect was not observed when only HRMECs were cultured (Figure 4B). To verify the changes in HRMEC permeability caused by pericytes or PS, tight junction proteins, including zona occludens-1 (ZO-1) and occludin, were assessed in HRMECs. Co-culture with pericytes led to increased expression of ZO-1 in HRMECs compared to that in co-culture with only HRMECs (Figures 4C,D). However, when PS was applied to the pericyte–HRMEC co-culture, ZO-1 expression in HRMECs decreased (Figures 4C,D). Despite these changes, occludin expression in HRMECs remained unaffected under the same conditions (Figures 4C,D). In addition, in pericyte–HRMEC co-culture, the increased permeability and reduced ZO-1 expression induced by PS were reversed when pretreated with the AKT activator SC79 (Figures 4E–G). Occludin expression in HRMECs remained unchanged under the same conditions (Figures 4F,G). However, in cultures with only HRMECs, neither PS nor the AKT activator SC79 affected permeability or ZO-1 and occludin expression (Supplementary Figures 5A–C). These findings indicate that PS triggers pericyte apoptosis, which reduces ZO-1 expression in endothelial cells, leading to increased endothelial permeability.

## 4 Discussion

The present study demonstrated that PS induces apoptosis in retinal endothelial cells and pericytes, albeit through distinct mechanisms. These findings align with those of previous studies reporting that PS promotes apoptosis in various cell types via oxidative stress or caspase-3 activation (42–46). For instance, PS-induced oxidative stress triggers apoptosis in human vascular endothelial EA.hy926 cells (47) and reduces the viability of human umbilical vein endothelial cells (48). Consistent with these reports, our data confirmed that PS exposure reduced viability and increased apoptosis in retinal endothelial cells (Figures 1A–C). However, unlike the oxidative stress-related mechanisms described in other models, PS did not induce oxidative stress in retinal endothelial cells (data not shown). Instead, apoptosis was mediated through inhibition of the PI3K/AKT and MAPK/ERK1/2 signaling pathways (Figures 2C,D). In HRMECs, PS suppressed both AKT and ERK1/2 phosphorylation, which was accompanied by increased levels of cleaved caspase-3 and Bax and decreased Bcl-2 expression, suggesting activation of the intrinsic apoptotic pathway. These molecular changes were reversed by treatment with AKT or ERK activators, confirming the causative role of these pathways in apoptosis (Figure 2D; Supplementary Figures 4A,B). In pericytes, PS reduced AKT phosphorylation without affecting ERK1/2, which was sufficient to increase cleaved caspase-3 and Bax levels, supporting a direct link between AKT inactivation and pericyte apoptosis. These findings underscore a tissue-specific mechanism of PS toxicity in retinal cells that operates independently of ROS. Given the pivotal role of pericyte loss in early diabetic retinopathy, our findings suggest that PS exposure compromises retinal microvascular stability and contributes to early pathological changes associated with disease progression.

Diabetic retinopathy is categorized into two primary types: non-proliferative and proliferative. Proliferative diabetic retinopathy indicates the presence of neovascularization or abnormal growth of blood vessels in the retinas (49). In the early stages, when neovascularization is absent, the condition is known as non-proliferative diabetic retinopathy (49). As the disease advances, it can progress to proliferative diabetic retinopathy, which is characterized by neovascularization and carries a high risk of severe vision problems (49). Retinopathy of prematurity is another type of proliferative retinopathy characterized by neovascularization or abnormal blood vessel growth in the retinas (50). In other words, promoting angiogenesis can trigger or worsen conditions such as proliferative diabetic retinopathy or retinopathy of prematurity. However, it was previously shown that PS inhibits tube formation in human umbilical vein endothelial cells (48). Similarly, in this study, PS reduced tube formation, migration, and proliferation in retinal endothelial cells, both under normal conditions and following treatment with angiogenic factors such as VEGF (Figures 3A–E). Therefore, although PS may not promote proliferative retinopathy, its pro-apoptotic and permeability-enhancing actions suggest that it contributes to non-proliferative retinopathy or exacerbate macular edema.

Pericytes are essential for maintaining endothelial tight junction integrity and preventing endothelial permeability (27). Pericyte loss is prominent in early diabetic retinopathy (51), which increases endothelial permeability and may lead to macular edema because of increased vascular leakage (27). This study provides evidence showing that PS may promote pericyte loss, thereby destabilizing endothelial junctions. A previous study demonstrated that PS directly reduces ZO-1 expression in brain microvascular endothelial cells and human vascular endothelial EA.hy926 cells (47, 52). However, PS did not directly affect ZO-1 expression or endothelial permeability (Figures 4B–D). Instead, PS decreased ZO-1 expression and increased endothelial permeability in HRMECs co-cultured with pericytes (Figures 4B–D). Moreover, when pericyte apoptosis was prevented using an AKT activator, the PS-induced loss of ZO-1 and increased endothelial permeability were prevented in co-culture systems (Figures 4E–G), highlighting that PS increases endothelial permeability primarily by inducing pericyte apoptosis, thereby disrupting BRB integrity.

We used 2 μm polystyrene particles, which fall within the microplastic size range (1 μm to 5 mm) and are commonly used in *in vitro* toxicity assessments. Although these particles are representative of commonly encountered environmental microplastics, they do not reflect the full diversity of particle sizes and shapes found *in vivo* (53). Notably, recent studies reported that polyethylene, polyethylene terephthalate, and polyvinyl chloride are more commonly detected in human tissues than in PS, and the concentrations used in our experiments (up to 20 μg/mL) exceed the highest concentrations measured in human blood (approximately 10 μg/mL (9);). This discrepancy reflects a limitation of the *in vitro* model and suggests that observed effects represent exaggerated responses compared to typical *in vivo* exposure. Moreover, a recent meta-analysis reported that micro- and nanoplastics were detected in cardiovascular and neurologically relevant tissues such as the heart, atherosclerotic plaques, brain, and olfactory bulb (12). Although these results support the potential for systemic distribution of microplastics and nanoplastics, they also emphasize that PS is not the most prevalent plastic type found in human tissues. Nonetheless, the present study provides valuable mechanistic insight into how microplastic exposure—particularly through PS—may disrupt retinal vascular integrity and highlights the need for further studies of plastic-related retinal toxicity.

In summary, we demonstrated that PS induces apoptosis in retinal endothelial cells by reducing PI3K/AKT and MAPK/ERK1/2 activity and in pericytes by inhibiting AKT signaling. These changes result in impaired angiogenic function and increased vascular permeability. Specifically, PS reduced the proliferation, migration, and tube formation of retinal endothelial cells and increased permeability by weakening tight junctions via pericyte apoptosis. Although PS does not appear to aggravate angiogenesis, it may contribute to the development or progression of retinopathy by promoting vascular leakage.

## Data Availability

The original contributions presented in the study are included in the article/Supplementary material, further inquiries can be directed to the corresponding author.
