# Supplementary material for: Polystyrene microplastics impair the function of human retinal microvascular endothelial cells and pericytes and increase vascular permeability in vitro
Source: Front Med (Lausanne). 2025 Jun 24;12:1587759. doi: 10.3389/fmed.2025.1587759 (PMC12234505; doi:10.3389/fmed.2025.1587759)
Supplement: Supplementary file 1 [file Data_Sheet_1.pdf]

Supplementary Material

Polystyrene microplastics impair the function of retinal cells and increase endothelial permeability *in vitro*

Jang-Hyuk Yun*

College of Veterinary Medicine and Institute of Veterinary Science, Kangwon National University, Chuncheon, Gangwon, 24341, Republic of Korea

***Address correspondence to: Jang-Hyuk Yun, D.V.M., Ph.D.**, College of Veterinary Medicine and Institute of Veterinary Science, Kangwon National University, Chuncheon, Gangwon, 24341, Republic of Korea. E-mail: yunjh@kangwon.ac.kr Phone: 82-33-250-8653 Fax: 82-33-259-5625

1.1 Supplementary Figures

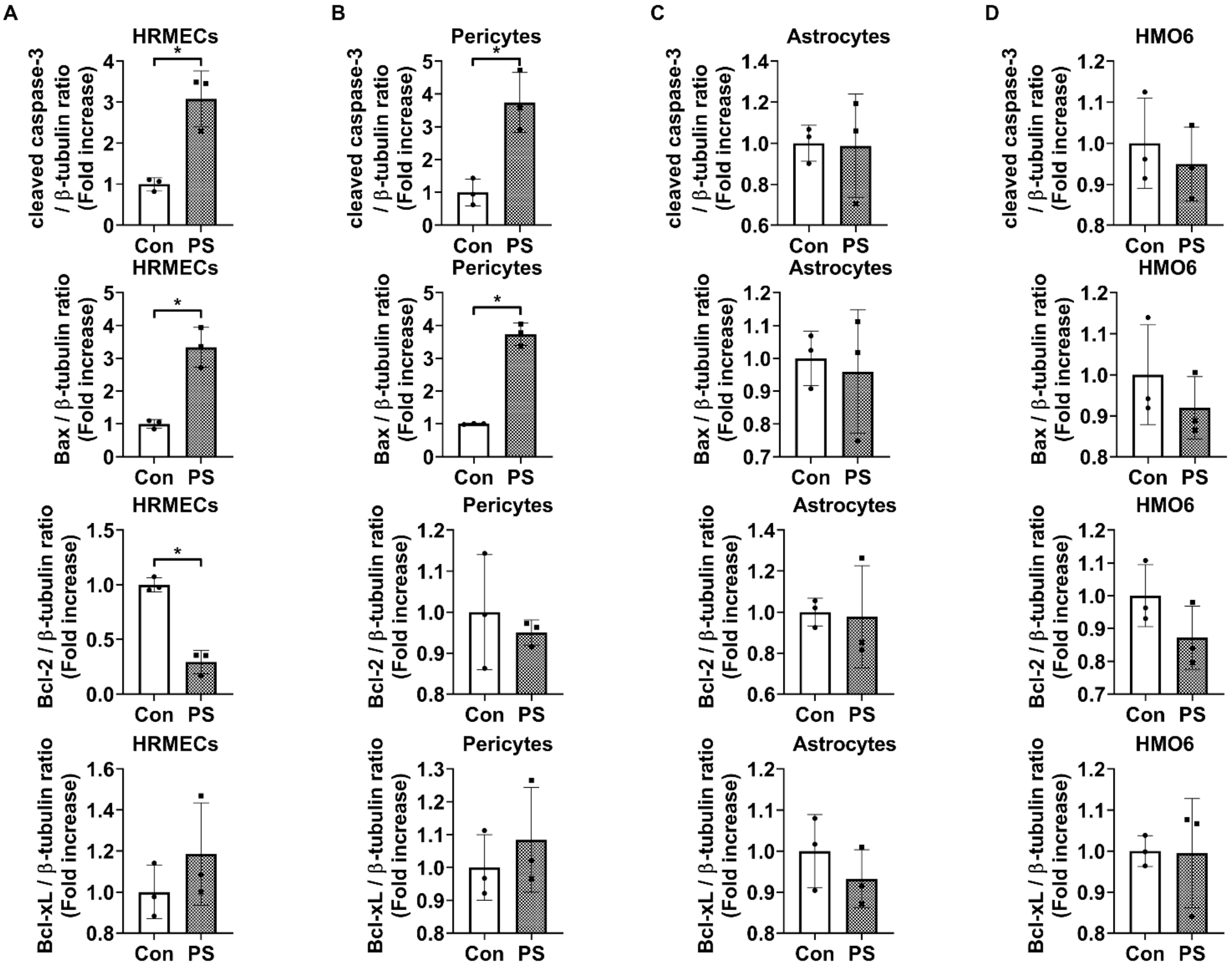

Supplementary Figure 1. PS induces apoptosis of retinal endothelial cells and pericytes. (A–D) Quantitative analysis of protein levels in Figure 1C was performed. Protein levels were calculated as the ratio of cleaved caspase-3, Bax, Bcl-2, or Bcl-xL to β-tubulin in (A) HRMECs, (B) pericytes, (C) astrocytes, and (D) HMO6 cells. ($n = 3$). *$P < 0.05$, determined using Student t test. HMO6, human microglial cell line; HRMEC, human primary retinal microvascular endothelial cell; PS, polystyrene.

A

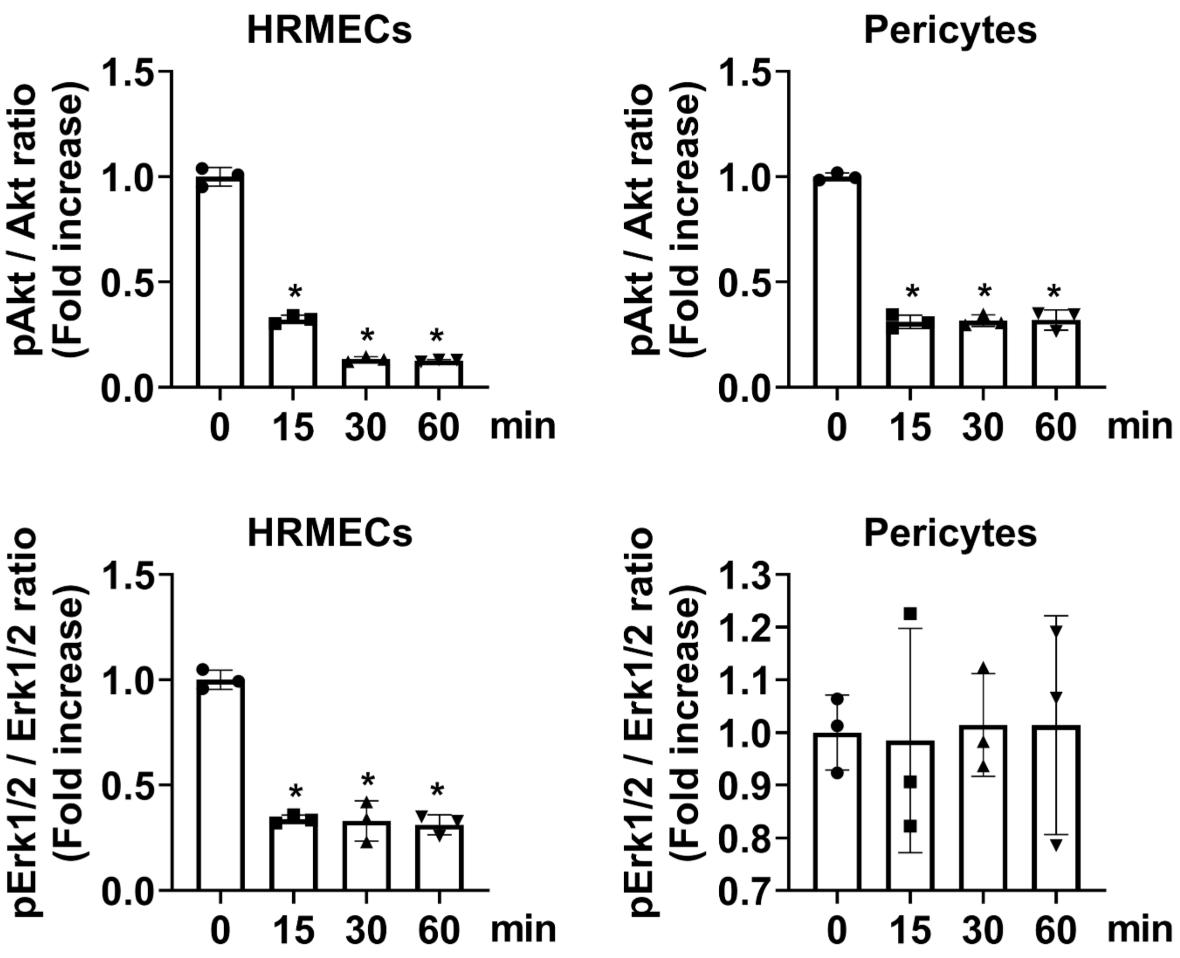

Supplementary Figure 2. PS reduces AKT or ERK1/2 phosphorylation in HRMECs and pericytes. (A) Quantitative analysis of protein phosphorylation shown in Figure 2A. Protein levels were calculated as the ratio of phosphorylated AKT (pAKT) to total AKT and phosphorylated ERK1/2 (pERK1/2) to total ERK1/2 in HRMECs and pericytes. Data represent the mean ± SD (n = 3). *$P < 0.05$, one-way ANOVA. ANOVA, analysis of variance; HRMEC, human primary retinal microvascular endothelial cell; PS, polystyrene.

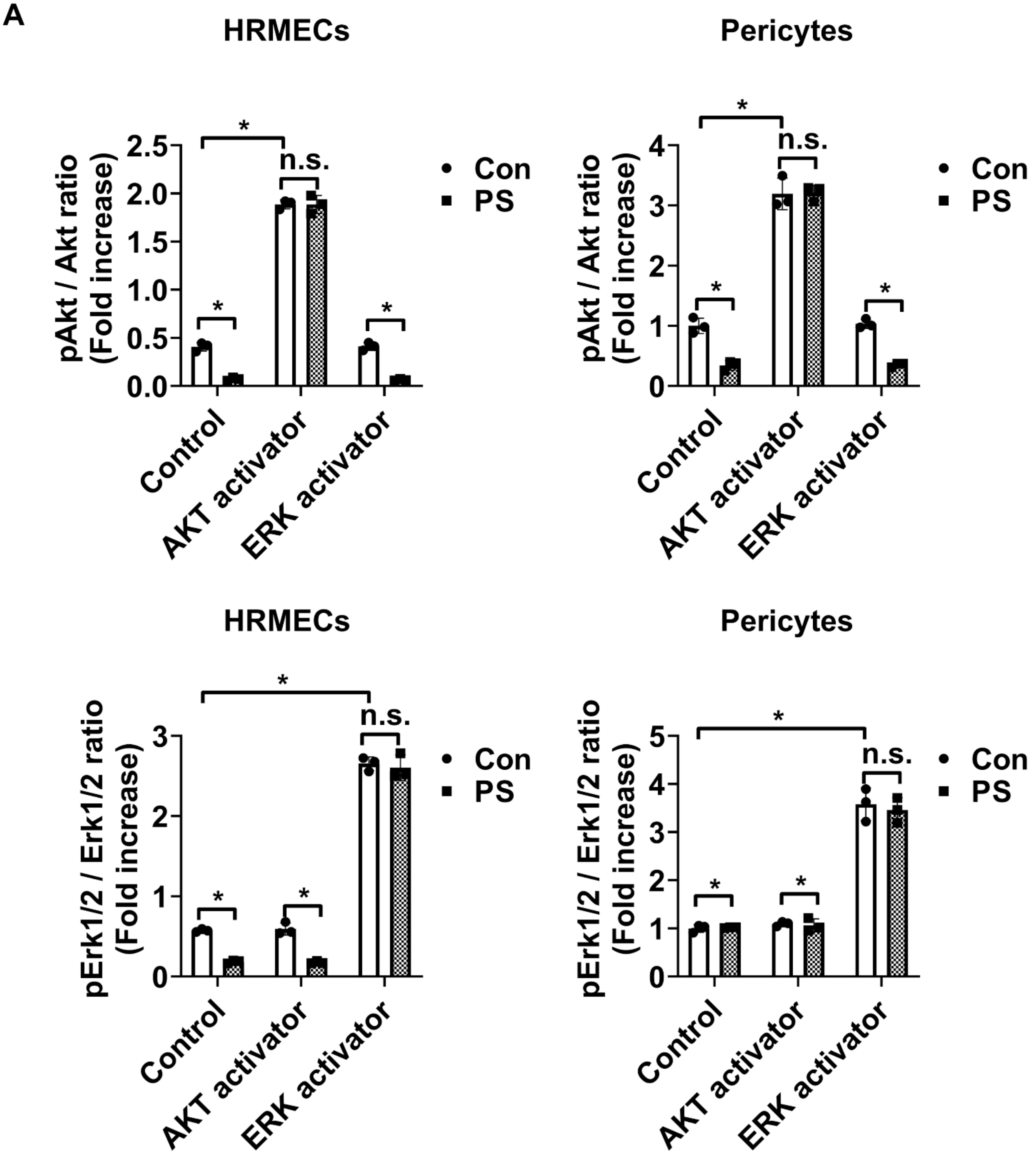

Supplementary Figure 3. AKT and ERK activators prevent PS-induced reduction in AKT or ERK1/2 phosphorylation. (A) Quantitative analysis of protein phosphorylation shown in Figure 2B. Protein levels were calculated as the ratio of phosphorylated AKT (pAKT) to total AKT and phosphorylated ERK1/2 (pERK1/2) to total ERK1/2 in HRMECs and pericytes. Data represent the mean ± SD (n = 3). n.s, not significant. *$P < 0.05$, two-way ANOVA. ANOVA, analysis of variance; HRMEC, human primary retinal microvascular endothelial cell; PS, polystyrene.

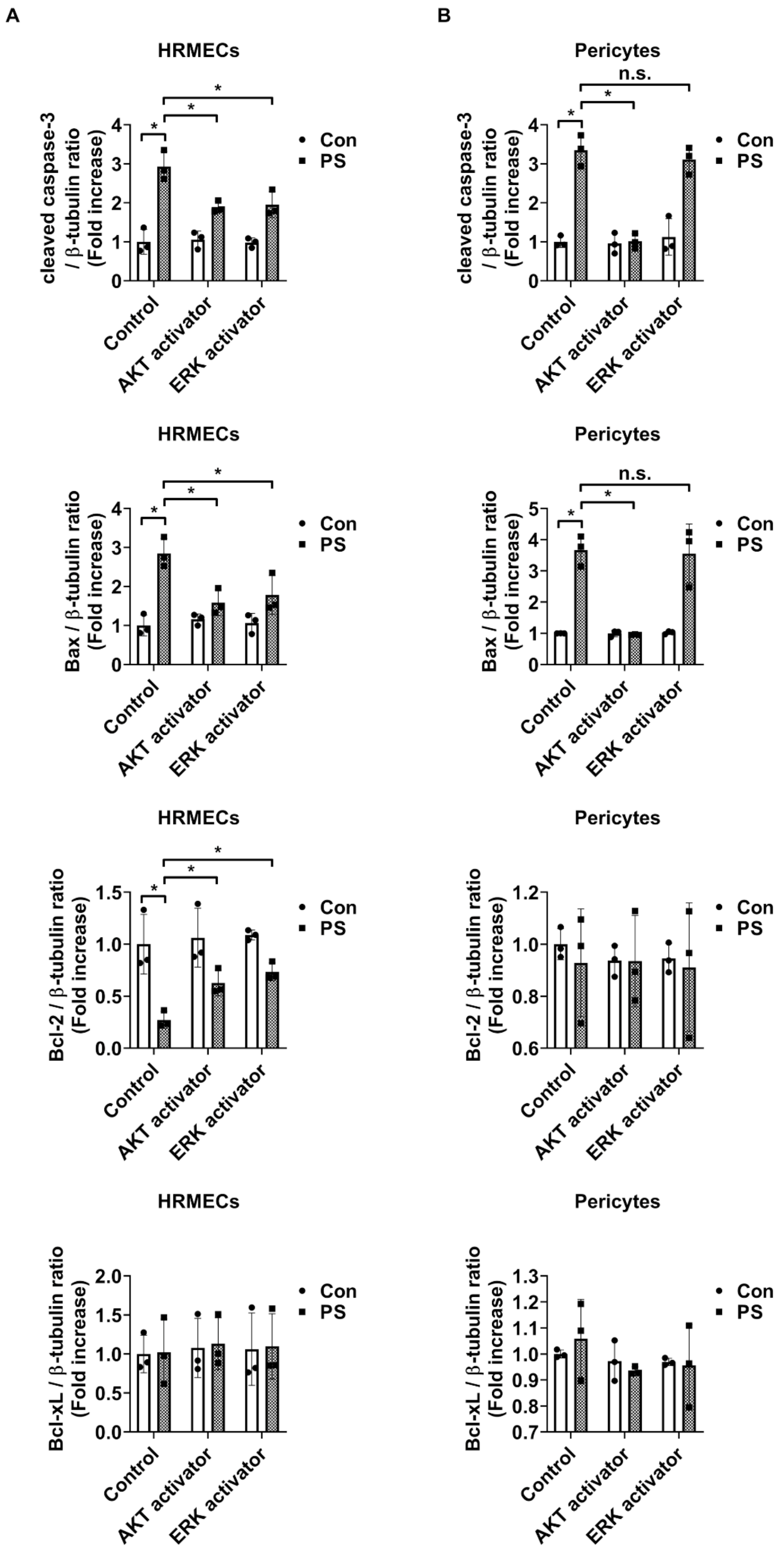

A
B
HRMECs
Pericytes
cleaved caspase-3 / β-tubulin ratio (Fold increase)
Bax / β-tubulin ratio (Fold increase)
Bcl-2 / β-tubulin ratio (Fold increase)
Bcl-xL / β-tubulin ratio (Fold increase)
Con
PS
Control
AKT activator
ERK activator
n.s.

Supplementary Figure 4. PS triggers apoptosis in retinal endothelial cells by lowering the activity of AKT and ERK1/2 and induces apoptosis in pericytes by lowering the activity of AKT. (A, B) Quantitative analysis of protein levels in Figure 2D. Protein levels were calculated as the ratio of cleaved caspase-3, Bax, Bcl-2, or Bcl-xL to β-tubulin in (A) HRMECs and (B) pericytes. ($n = 3$). n.s, not significant. *$P < 0.05$, two-way ANOVA. ANOVA, analysis of variance; HRMEC, human primary retinal microvascular endothelial cell; PS, polystyrene.

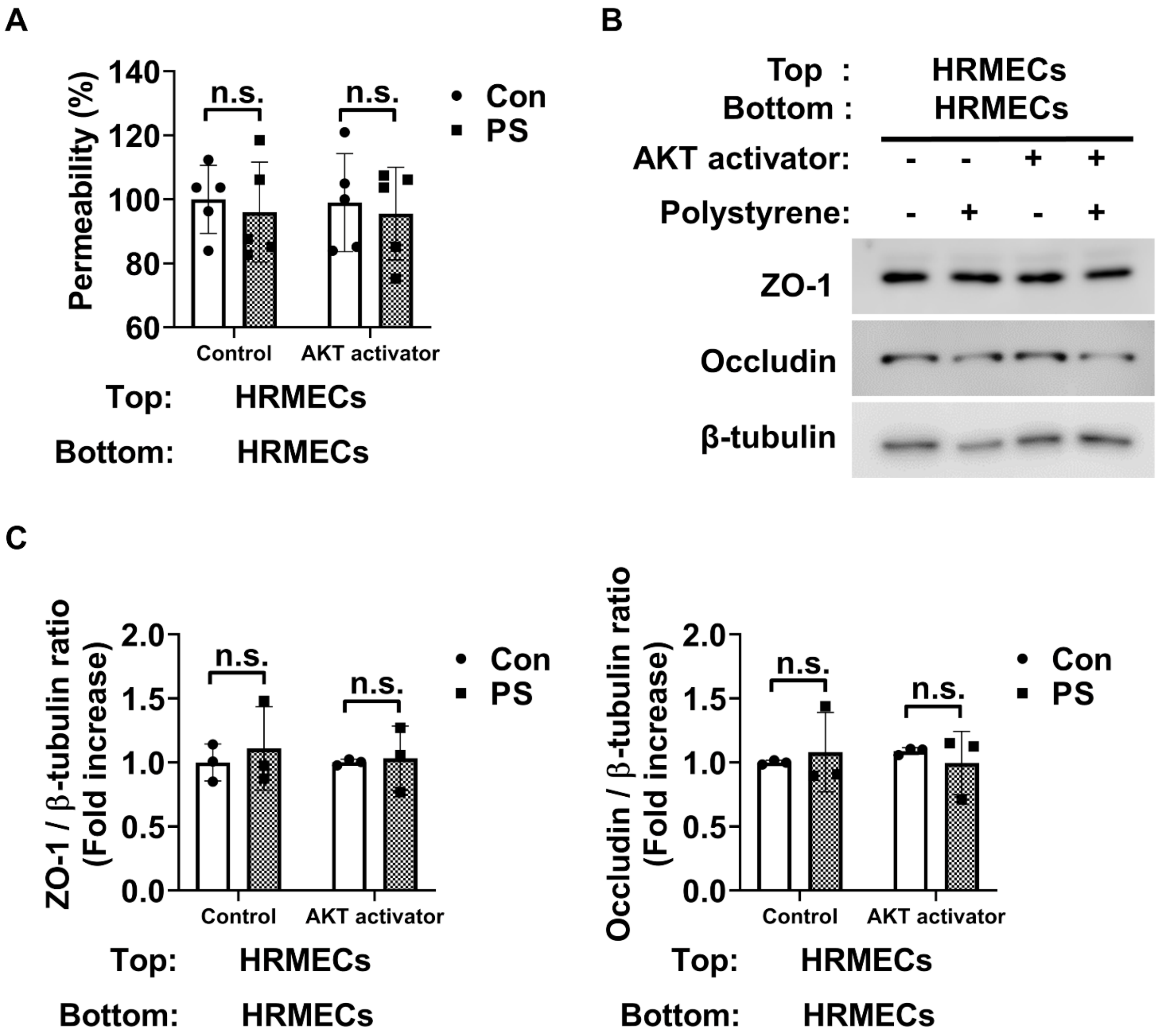

Supplementary Figure 5. PS and AKT activator SC79 do not directly affect the permeability of retinal endothelial cells. (A) Permeability was measured after treatment with PS with or without AKT activator SC79 (1 μg/mL) in co-cultured HRMECs (bottom) and HRMECs (top) (n = 5). n.s, not significant. two-way ANOVA. (B) Tight junction protein expression of ZO-1 and occludin was measured from the cell lysates of HRMECs on the top side of the Transwell obtained from (A). (C) Quantitative densitometric analysis in (B) to calculate the ratio of each protein to β-tubulin (n = 3). n.s, not significant. *P < 0.05, two-way ANOVA. ANOVA, analysis of variance; HRMEC, human primary retinal microvascular endothelial cell; PS, polystyrene.
